# The Role of Phosphatidylethanolamine Adducts in Modification of the Activity of Membrane Proteins under Oxidative Stress

**DOI:** 10.3390/molecules24244545

**Published:** 2019-12-12

**Authors:** Elena E. Pohl, Olga Jovanovic

**Affiliations:** Institute of Physiology, Pathophysiology and Biophysics, Department of Biomedical Sciences, University of Veterinary Medicine, Vienna A-1210, Austria

**Keywords:** reactive aldehydes, hydroxynonenal, oxononenal, free fatty acids, mitochondrial uncoupling protein, lipid bilayer membranes

## Abstract

Reactive oxygen species (ROS) and their derivatives, reactive aldehydes (RAs), have been implicated in the pathogenesis of many diseases, including metabolic, cardiovascular, and inflammatory disease. Understanding how RAs can modify the function of membrane proteins is critical for the design of therapeutic approaches in the above-mentioned pathologies. Over the last few decades, direct interactions of RA with proteins have been extensively studied. Yet, few studies have been performed on the modifications of membrane lipids arising from the interaction of RAs with the lipid amino group that leads to the formation of adducts. It is even less well understood how various multiple adducts affect the properties of the lipid membrane and those of embedded membrane proteins. In this short review, we discuss a crucial role of phosphatidylethanolamine (PE) and PE-derived adducts as mediators of RA effects on membrane proteins. We propose potential PE-mediated mechanisms that explain the modulation of membrane properties and the functions of membrane transporters, channels, receptors, and enzymes. We aim to highlight this new area of research and to encourage a more nuanced investigation of the complex nature of the new lipid-mediated mechanism in the modification of membrane protein function under oxidative stress.

## 1. Reactive Oxygen Species and Their Derivatives, Reactive Aldehydes

With regard to reactive oxygen species (ROS), these include unstable short-lived molecules that contain oxygen (O_2_**^.^**, H_2_O_2_, and OH**^−^**) and are highly reactive in cells. The term “ROS” is often substituted by the phrase “free radicals”; however, strictly speaking, only O_2_**^.^** and OH**^-^**are considered free radicals. Besides oxygen, reactive species (RS) may contain nitrogen, carbon, sulfur, and halogens. 

**Figure 1:**
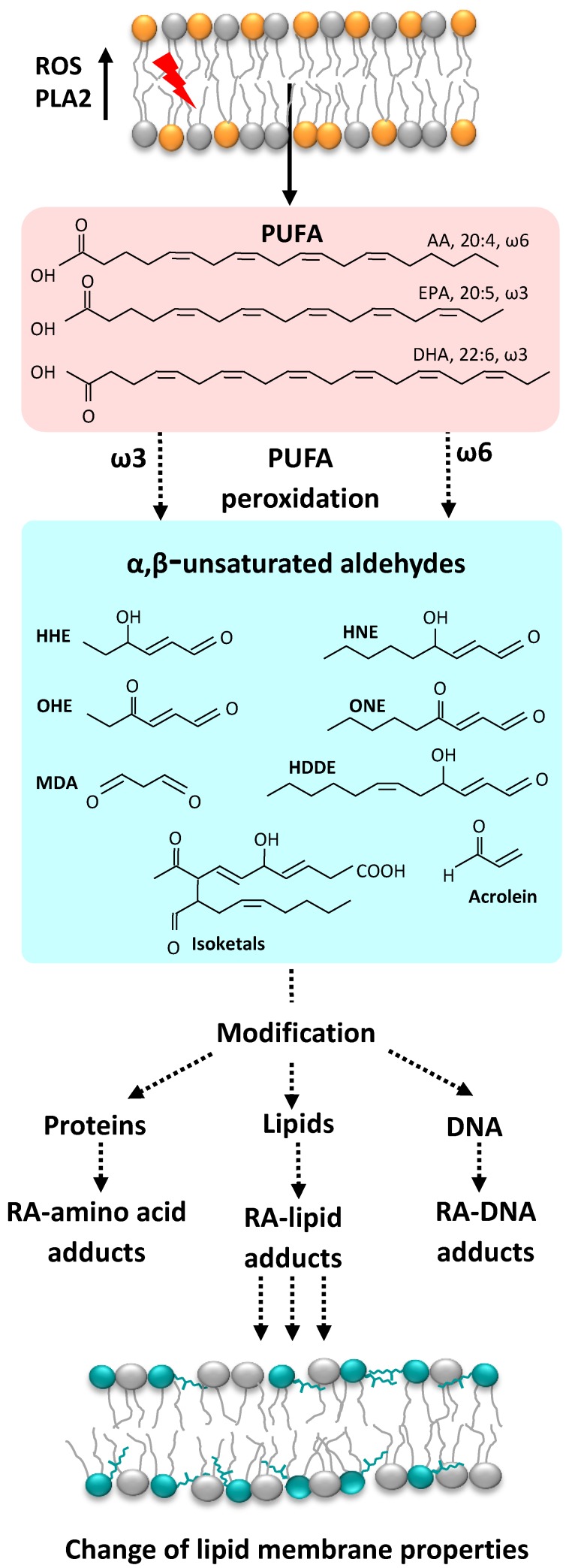
Elevated levels of ROS induce PUFA peroxidation in the cell membrane and the formation of different α, β-unsaturated RAs which can react with proteins, lipids, and DNA. Abbreviations: AA, arachidonic acid; DHA, docosahexaenoic acid; EPA, eicosapentaenoic acid; HDDE, 4-hydroxydodeca-(2E,6Z)-dienal; PLA2, phospholipase A2; PUFA, polyunsaturated fatty acid.

**Figure 2:**
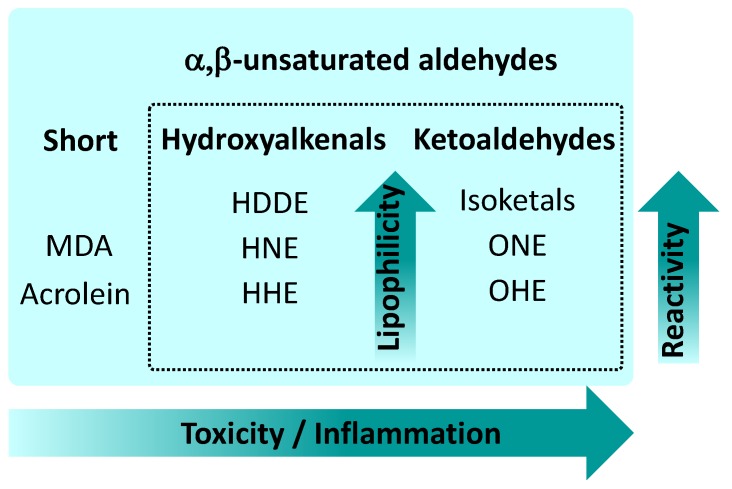
Main types of α,β-unsaturated aldehydes and relations between their lipophilicity, reactivity, and toxicity. Abbreviations as used in Figure 1.

Over 90% of ROS in eukaryotic cells are produced by mitochondria [1]. Mitochondria produce a substantial amount of superoxide anion at Complex I and via autoxidation of a ubisemiquinone anion radical at Complex III, where O_2_**^.^** is released on both sides of the membrane (for recent reviews see [2,3,4,5]). Superoxide anion may give rise to a variety of reactive carbonyl species (RCS, reactive aldehydes (RAs)), which are three to nine carbons in length (Figure 1). Of these, α,β-unsaturated aldehydes (4-hydroxy-trans-2-nonenal (HNE) and acrolein), di-aldehydes (malondialdehyde (MDA) and glyoxal), and keto-aldehydes (4-oxo-trans-2-nonenal (ONE) and isoketals (IsoK)) are the most toxic RAs (Figure 2).

RAs can be more destructive than ROS because (1) they have a much longer half-life (i.e., minutes to hours instead of microseconds to nanoseconds for most free radicals), and (2) the non-charged structure of aldehydes allows them to migrate long distances from the production site through hydrophobic membranes [6,7,8]. Reactivity between aldehydes differs both qualitatively and quantitatively. The most extensively studied aldehyde, HNE is generated by the oxidation of lipids containing polyunsaturated omega-6 acyl groups, such as arachidonic or linoleic groups, and of the corresponding fatty acids (FAs). This aldehyde elicits deleterious effects primarily by oxidizing intracellular components, including DNA, lipids, and proteins [9]. Another aldehyde, 4-oxo-2-hexenal (OHE), is generated by the oxidation of ω-3 polyunsaturated FAs, which are commonly found in dietary fish oil and soybean oil [10]. OHE is thought to possess DNA-damaging potential similar to that of HNE. In contrast, ONE was shown to be a more reactive protein modifier and cross-linking agent than HNE [11]. It has been proposed that the greater neurotoxicity of ONE may indicate different reactivity characteristics than those of HNE. In comparison, MDA possesses a stronger mutagenic and carcinogenic potential in mammalian cells than HNE [12]. 

The estimation of exact RA concentrations in cells is difficult since concentrations of several µM, the maximum reported for the cell cytoplasm, are averaged values. However, it is plausible that RA concentration levels may be much higher locally for a short period of time [13]. Whereas cellular concentrations of HNE under physiological conditions can reach 0.3 mM, HNE accumulates at concentrations up to 5 mM in cellular membranes under conditions of oxidative stress [14]. Also, Esterbauer et al. suggested that HNE and other aldehydes are unlikely to reach physiological concentrations of approximately 100 μM [7]. However, much higher levels may be transiently achieved in the vicinity of peroxidizing membranes because of the high lipophilicity of RA. As an example, within the lipid bilayer of isolated peroxidizing microsomes, the concentration HNE is approximately 4.5 mM [13,15]. The concentration of an RA in the membrane is strongly dependent on its lipophilicity and, for example, is higher for ONE and HNE than for 4-hydroxy-2-hexenal (HHE) [16].

## 2. ROS Detoxification Systems 

The cell’s own systems of defense from oxidative stress include different ROS detoxification systems, such as superoxide dismutase, catalase, and glutathione peroxidase (for detailed reviews see [12,17]). Several lipophilic or water-soluble, membrane-permeable molecules (tocopherol, carotenoids, anthocyanins, polyphenols, and uric and ascorbic acid [18]) can work as endogenous or nutritional antioxidants (for review see [19]).

The production of ROS in mitochondria is also sensitive to the proton motive force and may be decreased by artificial uncouplers (e.g., dinitrophenol, carbonylcyanid-*m*-chlorphenylhydrazon (CCCP), carbonilcyanide *p*-triflouromethoxyphenylhydrazone (FCCP)) or proteins transporting protons from the intermembrane space to the matrix, a process termed “mild uncoupling” (Skulachev, 1998). 

Several members of a mitochondrial membrane protein superfamily, known as the solute carrier family (SLC25), such as adenine nucleotide transporter (ANT) [20,21], dicarboxylate carrier [22], phosphate, aspartate glutamate carrier [23], and members of the uncoupling protein subfamily (UCP1–4) were proposed to be involved in proton transport. 

It is well accepted that the best investigated member of the UCP family—UCP1—uncouples substrate oxidation from mitochondrial ATP synthesis by transporting protons from the intermembrane space to the matrix [24,25,26,27,28,29]. The protonophoric ability of other uncoupling proteins is a longstanding issue in controversial debates [30]. The proton-transporting capacity of UCP2 and UCP3 was shown to be comparable with the capacity of UCP1 in lipid bilayer membranes reconstituted with recombinant proteins (2–14/s, for a review see [31]). Several studies in multiscale and biomimetic systems indicate that the function of UCP2/UCP3 is associated with the transport of different metabolic substrates [32,33,34], possibly alongside proton transport [26,35,36,37]. A recent comparison of wild type and UCP2^-/-^ or UCP3^-/-^ knockout mice [21] implied that both proteins are not involved in H^+^ transport. Unfortunately, the tissue(s) chosen in this study were previously shown to have no presence or very low abundance of UCP2/UCP3 under physiological conditions [38,39]. 

Nègre-Salvayre et al. was the first to suggest UCP2 involvement in ROS regulation, combining the largely accepted view that a high mitochondrial membrane potential leads to increased production of ROS (particularly superoxide anion) with the idea that UCP2 diminishes the membrane potential by transporting proteins from intermembrane space to the mitochondrial matrix [40]. This suggestion was extended by Brand and colleagues who proposed that UCP activity was regulated by superoxide [41] or RAs (HNE) [42]. A coherent theory for a feedback mechanism was formulated whereby increased ROS production would activate uncoupling to decrease ROS formation; oxidative damage would thereby be reduced (for review see [43]). In contrast, Cannon’s group, using brown-fat mitochondria from UCP1^-/-^ and superoxide dismutase (SOD^-/-^) knock-out mice, showed that HNE could neither (re)activate purine nucleotide-inhibited UCP1, nor induce the additional activation of innately active UCP1 [44]. No support for the tenet that superoxide directly or indirectly regulates UCP1–UCP3 activity could be found [45,46]. In subsequent studies, a high membrane potential was suggested as a requirement for the activation of UCP-mediated uncoupling by HNE [47]. Experiments performed in a well-defined system of bilayer membranes reconstituted with recombinant UCPs [48] revealed that HNE did not directly activate either UCP1 or UCP2. However, HNE strongly potentiated the membrane proton conductance increase mediated by different long-chain FAs in UCP-containing and UCP-free membranes. These results contributed to an understanding of the controversial results observed by different groups in multiscale systems and allowed to investigate the molecular mechanism of HNE–UCP interactions (see Section 4.4).

## 3. Mechanisms of RA Action

A consensus exists that RAs play a dual role in cellular processes: They are known to modify proteins [49,50,51], DNA [52,53], and lipids [16,54], but are also involved in important signaling pathways [50]. However, the molecular mechanisms of their action are still far from being well understood. It is becoming increasingly clear that in both cases, RAs form adducts with the nucleophilic groups of proteins, DNA and lipids [55]. Meanwhile, it is evident that RAs not only target a large variety of molecules, but also that the mechanisms of such interactions differ. The latter are still poorly understood and seem to depend on the chemical structure of RAs, interaction molecules, lipid environment, and the distance between the RA source and the target molecule [56].

An increasing number of studies have shown that RAs bind to proteins and impair their function by modification of amino acid residues and protein crosslinking to an extent that depends on their reactivity (for review see [14,57]). Both ONE and HNE covalently bind to cysteine, histidine, and lysine, while ONE also binds to arginines. The reactivity of HNE (k_HNE_) toward amino acids was reported to be: cysteine (1.21 M^−1^ s^−1^) >> histidine (2.14 × 10^−3^ M^−1^ s^−1^) > lysine (1.33 × 10^−3^ M^−1^ s^−1^) [58]. This means that the reactivity of thiol group-containing cysteines is higher than that of amino group-containing lysines and histidines. It further implies that HNE and ONE would primarily attack the thiol groups of proteins disabling disulfide bridges formation and affecting thereby protein function(s) [59]. ONE was reported to be more reactive than HNE, given k_ONE_/k_HNE_: cysteine 153 >> histidine 10.3 > lysine 5.61. In contrast, extremely reactive IsoK rapidly reacts with positively charged lysine residues rather than with thiols [60]. The modification and crosslinking of amino acid residues, proteins, and peptides are perceived as major toxic effects of RA. The selective and reversible oxidation of key residues in proteins that presumably leads to conformational changes and the alteration of protein activity and function [61,62] is a physiological mechanism well-studied in cytosolic (hydrophilic) proteins.

An important role for the membrane lipid, phosphatidylethanolamine (PE), in the functions of cell membranes and transmembrane proteins (discussed in Section 4.2) implies that its modification affects different processes in the cell. Whereas rate constants for the reaction of HNE with amino acids have been intensively studied, no binding kinetic data exists concerning the reaction rate of HNE with amino groups of lipids (PE, phosphatidylserine (PS), and sphingomyelin (SML)). However, the interaction with amino groups of lipids seems to be highly relevant for membrane proteins, especially in membranes with a low protein/lipid ratio (e.g., oligodendrocytes). Previously it was shown that the function of membrane uncoupling proteins is altered only in the presence of PE [16], although western blot analysis revealed that HNE was also bound to cysteines [48].

## 4. Phosphatidylethanolamine as a Crucial Target for Reactive Aldehydes

### 4.1. Phosphatidylethanolamine and Its Physiological Functions

Phosphatidylethanolamine (PE) is the second most abundant phospholipid, after phosphatidylcholine (PC), in the membranes of all mammalian cells. On average, it makes up 25% of the total phospholipid mass [63]. The highest amount of PE, up 45% of all phospholipids, is found in the membranes of tissues of the neuronal system, such as white matter of the brain, nerves, and spinal cord [64]. PE is a non-bilayer lipid, more abundant in the inner than in the outer leaflet of the cell membranes [65]. Due to its conical shape, PE modulates membrane curvature and lateral pressure [66,67] and thus supports membrane fusion [68,69,70,71] and function of several membrane proteins [67,71,72].

PE is a fundamental component of biological membranes, needed for many cellular functions. Besides being a precursor for other lipids [73], PE is involved in a multitude of physiological functions. Among others, PE (1) supports chaperoning membrane proteins to their folded state [74], (2) activates oxidative phosphorylation [75,76], (3) is involved in apoptotic [77] and ferroptotic [78] cell death pathways, (4) mediates the modification of prions from a nontoxic to toxic conformation [79], and (5) is crucial for the synthesis of glycosylphosphatidylinositol-anchored proteins essential for cell viability [80]. The importance of PE for cell function is evident in the existence of four separate PE biosynthetic pathways [81], one of which takes place in the inner mitochondrial membrane [76]. 

Disorders in PE metabolism have been implicated in many chronic diseases, such as Alzheimer’s disease, Parkinson’s disease, and nonalcoholic liver disease [82], as well as metabolic disorders such as atherosclerosis, insulin resistance, and obesity [63]. Increased levels of PE have been described in cancer cells leading to PE being regarded as a target in the development of anticancer therapies [83].

### 4.2. PE Adducts 

**Figure 3:**
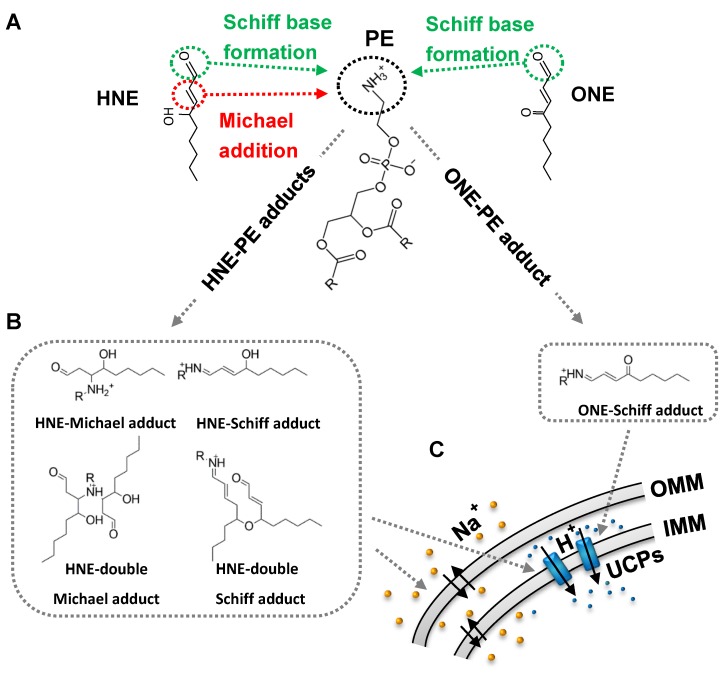
Mechanisms of modification of the phosphatidylethanolamine (PE) by RAs and their impact on function of mitochondrial and other cell membranes. HNE and ONE covalently bind to the PE primary amine group (**A**), forming different RA-PE adducts (Michael or Schiff base type) (**B**). HNE-PE adducts in lipid bilayer membrane decrease free energy barrier ∆G and increase permeability for cations. Localization of ONE-PE and HNE-PE adducts in the cellular membrane change bending properties and lateral pressure profile of the membrane that results in increased proton translocation mediated by uncoupling protein (UCPs) (**C**). Abbreviations: OMM, outer mitochondrial membrane; IMM, inner mitochondrial membrane.

To date, only a few groups have studied the ability of reactive aldehydes (RAs) to modify the headgroup of amino-phospholipids (amino-PLs), predominantly PE, and characterized formed adducts. Reactions of α,β-unsaturated aldehydes (HHE, HNE, and ONE) with amino-PLs lead to the formation of different adducts, such as Michael adducts (MAs) and Schiff base adducts (SBs) (Figure 3). Depending on experimental conditions (for example, incubation) more complex types of adducts, such as double-MAs, double-SBs, and pyrrole adducts, may be formed [84,85,86,87,88,89]. Initially, studies of modifications of amino-PLs by α,β-unsaturated aldehydes and hydroxyalkenals, and subsequently ketoaldehydes (IsoLGs) and short- and long-chain aldehydes, were performed. Recently, it was demonstrated that primary amines can react with glucose [90] and amide linkages [91], and modify the head group of amino-PLs in a similar manner (s. below). 

The first described covalent modifications of the lipid head group by an RA were the reactions of the HNE with PE and PS. As the main products, PE-MAs and PS-MAs were identified. Imine and pyrrole adducts were detected only in PE, but to a much lesser extent [84]. Other authors reported covalent modification of the PE headgroup by long chain saturated alkenals (e.g., pentadecanal, heptadecenal), and α-hydroxyalkenals (α-hydroxyhexadecanal, α-hydroxyoctadecanal), produced during oxidation of plasmalogen, resulting in PE-SB adducts, also known as *N*-alkyl-PEs [92,93,94].

Evaluation of the role of the acyl chain length of α,β unsaturated hydroxyalkenals (4-HHE, 4-HNE and 4-HDDE) on their ability to covalently modify different types of PEs revealed (1) a correlation between their reactivity to PE with their increasing hydrophobicity in the order HHDE > HNE > HHE (Figure 2), and (2) their selectivity towards different PEs: all three hydroxyalkenals favored modification of plasmalogen-PE over other PEs [85]. 

Comparison of the covalent modification of PE due to a reaction with HNE and the more toxic ketoaldehyde, ONE, which has the same length but with a carbonyl instead of a hydroxyl group on C4, revealed that this difference led to the formation of different adducts (Figure 3, B). While HNE formed four types of PE-adducts (MAs, SBs, double-MAs, and double-SBs), only one ONE-PE adduct (SB type) was detected [16]. These results highlight how the toxicity of ONE can be explained by the formation of only one type of ONE-PE adduct compared to a joint effect of several types of HNE-PE adducts. 

With increased lipophilicity and complexity, the reactivity of ketoaldehydes also increases (Figure 2). Reactive γ-ketoaldehydes (γKA), also named IsoK or isolevuglandins, are peroxidation products of arachidonic acid formed via the isoprostane pathway [60]. In vitro experiments have revealed that IsoK covalently modified the PE headgroup at a higher rate than the well-characterized HNE, forming IsoK-PE SBs and IsoK-PE pyrrole adducts [95]. Further, it was shown that the reaction rate of IsoK with PE is significantly higher than those with protein or DNA [96]. Recent evidence indicates that IsoK-PE adducts act as inflammatory mediators in the cell [97,98]. However, the molecular mechanisms are largely unknown. One can speculate that many of the effects previously attributed to protein modification due to IsoKs [99,100] could, in fact, be due to their ability to modify PE. It has been shown that even more simple products of arachidonate oxidation, such as diverse carboxyacyls, chemically react with the PE amine group, making a family of so-called amide-linked PEs and forming predominantly SBs and the pyrrole type of adducts. These adducts have been implicated in the inflammation of endothelial cells [91,94]. 

In addition, the “smallest” aldehydes, such as MDA and acrolein, are capable of modifying the PE headgroup through the initial formation of SB, ending in more complex products. For example, the predominant product of the incubation MDA and PE was identified as dihydropyridine-PE (DHP-PE) [91], while two acroleins in reactions with PE formed a compound termed (3-formyl-4-hydroxy)-piperidine-PE (FDP-PE) [101]. The involvement of MDA-PE and acrolein-PE adducts in the inflammatory process is moderate compared to HNE- or IsoK-PE adducts [91]. Due to their higher hydrophilicity, such RAs are thought to easily leave the lipid membrane and react with cytosolic proteins to a greater extent than with membrane lipids. 

It should be mentioned that the PE amine headgroup can be covalently modified by glucose and several fungal products. Glucose has an aldehyde group that can react with the primary amine of aminophospholipids via Maillard reactions to form Amadori adducts, (e.g., glucose phosphatidylethanolamine (gPE) and glucose phosphatidylserine (gPS)) [90,102,103]. Under conditions of oxidative stress, Amadori adducts undergo degradation to form advanced glycation products (goxPE) [104]. Several authors suggest gPEs and goxPEs are involved in diabetic and related neurodegenerative diseases [105,106]. Ophiobolin A (OPA) is a compound found in a fungus that is toxic to plant cells. OPA reacts with the primary amine of PE and forms pyrrole-containing OPA-PE adducts that show cytotoxic effects on some cancer cells [83].

### 4.3. Modification of Membrane Properties by PE and PE Adducts

Because of its conical shape, PE is essential for the processes of membrane budding, fission, and fusion [64,107,108]. In the lipid bilayer membrane, PE affects a lateral pressure profile and modulates membrane curvature; together with other lipids, PE provides an environment for optimal conformation and function of transmembrane proteins [67]. 

Although different studies have reported that RAs form adducts with aminophospholipids, their impact on the lipid bilayer membrane has been poorly studied. Recently, Jovanovic et al. [16] showed that PE adducts, formed after incubation of PC/PE lipid membranes with α,β-unsaturated aldehydes, significantly increased negative membrane ζ-potential in the order HHE < HNE << ONE. Notably, RAs did not influence the ζ-potential in PC lipid bilayers. An evaluation of the influence of PE adducts on the order parameter, S, revealed that only modification of PE by ONE leads to an increase in the bilayers’ fluidity, caused by alterations in the spatial arrangement of aliphatic chains in the lipid membrane. In contrast, HHE-PE adducts and HNE-PE adducts did not change the order parameter. Covalent modification of PE by HNE increased sodium permeability across the phospholipid bilayer by four orders of magnitude, while in the absence of PE the effect was not observed [89]. A calculation of the Nernst potential in the presence of a proton gradient revealed that the HNE-mediated total membrane conductance, G_m_, in PE-containing lipid membranes was mainly caused by cations (2/3 G_m_) rather than by protons (1/3 G_m_). Surprisingly, this effect was not recorded for the more toxic ONE. Molecular dynamic (MD) simulations of a lipid bilayer membrane composed of PC and either HNE or ONE adducts suggested that all types of HNE-PE adducts (especially the double adducts, D-SB-HNE and D-MA-HNE) became anchored deeper in the hydrophobic region, while ONE-PE adducts were entirely localized in the headgroup region of the lipid membrane. Study of the structural properties of the lipid bilayer revealed that double HNE adducts caused an increase in the area per lipid and a decrease in hydrophobic core thickness. The decrease of lipid dipoles per unit surface area diminishes membrane dipole potential [109]. As a consequence, the free energy barrier (∆G) for cations should decrease [110]. In turn, the permeability for sodium ions was increased [89]. 

Guo et al. [94] measured T_H_-shifts in the bilayer to a hexagonal phase transition temperature of DiPoPE (T_H_) incubated with 4-oxo-pentanal (OPA), γKA and glutaryl (glt) using differential scanning calorimetry to prove whether the formation of such adducts altered the curvature of the lipid membrane. γKA-PE and OPA-PE adducts showed similar behavior, and increased negative membrane curvature, while an *N*-glt-PE adduct showed the opposite effect to promote a positive membrane curvature. The observed change in the membrane curvature was consistent with the suggested localization of PE adducts. While γKA-PE and OPA-PE adducts are supposedly localized in the hydrophobic region, the *N*-glt-PE adduct is localized in the headgroup region. These results confirmed the assumption that modification of the PE headgroup alters lipid bilayer membrane properties, such as membrane curvature and, consequently, lateral pressure profile. 

Modification of the lipid shape due to the formation of ONE-PE adducts was reported to affect membrane curvature, which then altered the elastic properties of the lipid bilayer and the lateral pressure profile [111]. In general, due to the difference in RA-PE adduct distribution between the two leaflets, asymmetric changes of spontaneous membrane curvature may arise. In turn, the stability of membrane domains (lipid rafts) may be altered [112].

### 4.4. Modification of Membrane Transporter Function in the Presence of PE Adducts 

Both proteins and lipids were identified as targets of RA activity. However, while modification of cytosolic proteins by the activity of RAs has been extensively studied, and is directly related to protein dysfunction [49], an investigation of the impact of RAs on transmembrane proteins has only been made to a very modest extent, mostly due to their hydrophobicity. The observed alteration of membrane protein function was interpreted in the same way, assuming a direct connection between the modification of certain amino acid residues and protein function. 

The investigation of RA–protein interactions using artificial lipid membranes reconstituted with several transporters (mitochondrial transporter UCP1, potassium transporter valinomycin and uncoupler CCCP) surprisingly demonstrated that RA altered the transport activity of these molecules only when in the presence of the PE [16]. The greatest effect was elicited by ONE, which was more toxic in cell experiments, followed by HNE. HHE showed a much weaker effect, probably due to its lower hydrophobicity. Experiments further revealed that covalent modification of the PE headgroup causes changes in the electrical and mechanical properties of the lipid membrane, such as the boundary potential, order parameter and membrane bending rigidity [111,113]. According to MD simulations, the position of the RA-PE adduct in the lipid bilayer was responsible for the observed changes. Similar to the dipole potential modifier, phloretin [114], ONE- and HNE-PE adducts altered the boundary potential in the lipid membrane, and decreased the positive membrane energy barrier [115]. This resulted in increased valinomycin-mediated potassium transport and decreased proton transport mediated by CCCP. The same molecular mechanism could not explain the RA-PE action on UCP1 since UCP1-mediated proton conductance was not affected in the presence of dipole potential modifiers. However, MD simulations suggested that formation of RA-PE adducts change the form of PE from an originally negative intrinsic curvature to the opposite one, which was confirmed by observed changes of membrane bending rigidity [116]. Notably, a decrease of membrane bending rigidity in the presence of RA-PE adducts was in the order ONE > HNE > HHE, consistent with their effect on UCP1. The change in membrane curvature by the formation of RA-PEs, and related changes in the membrane lateral pressure profile, were made responsible for the modification of UCP1 transport function. In contrast to that previously shown for cytosolic proteins, modification of UCP1 and UCP2 by RAs [16,48] cannot activate the proteins directly, but rather by a described novel PE-mediated mechanism. 

Interestingly, glycated and glycoxidized PEs also alter the transport function of valinomycin in the same direction as RA-PEs, but to a more moderate extent [90]. PE glycation led to a similar change in negative membrane surface potential, as shown for RA-PEs. It indicates that glycated and glycoxidized PEs may decrease the positive energy membrane barrier in the lipid bilayer for cations comparable to the membrane dipole modifier, phloretin [114] and RA-PEs. An observed change in melting temperature upon PE glycation indicates a change in the membrane curvature [90], which allows us to hypothesize that such glucose-derived modifications on PEs could also affect the function of transmembrane proteins.

Unfortunately, we didn’t find examples demonstrating the impact of PE adducts on other transmembrane proteins than UCPs [16,48,90]. Although few groups demonstrated the modifications of the PE in cells and tissues [91,94,95,99], the possible impact of PE-adducts on the function of the membrane proteins was neither studied nor discussed. Guided by the hypothesis that the formation of RA-PE adducts could be involved in the pathogenesis of diseases associated with oxidative damage, authors focused on their involvement in the signaling and inflammatory processes. Considering the emerging role of lipid shape and membrane curvature on the function of transmembrane proteins, as well as their distribution in the membrane [117,118], the modification of PEs and their impact on the other membrane proteins have to be seriously studied.

## 5. Conclusions and Outlook

The question of how the functions of membrane transporters are modified under oxidative stress is a central issue that remains unexplained at the molecular level. ROS and their derivatives, RAs, are implicated in many diseases and, furthermore, in many signaling pathways. Current research has mainly focused on the aldehyde-mediated modification of protein amino acids, such as cysteine, lysine, and histidine, which supposedly affects the conformation of proteins. Recently, it was hypothesized that this mechanism may be more relevant for cytosolic proteins [16]. In contrast, the mechanism by which RAs modify the functions of membrane proteins may fundamentally differ from that of hydrophilic proteins. We have recently demonstrated that the initial binding of aldehydes to PE is a crucial step for alteration of the RA-mediated activity of different membrane transporters, such as mitochondrial inner membrane UCP1, the ionophore valinomycin, and the protonophore CCCP [16]. A lipid-mediated mechanism seems to be even more relevant for membranes abundant in PE, PS, or SML (e.g., mitochondria, bacteria) and for membranes with a low protein/lipid ratio, such as the membranes of oligodendrocytes. 

Whereas one can argue that short- and middle-chain aldehydes have approximately equal affinity in binding to the primary amine of an amino phospholipid or amino acid, very reactive long chain IsoK (products of the AA, 20:4, ω-6) bind to PE at a significantly higher rate than to proteins or DNA due to their strong hydrophobicity, as already experimentally shown [96]. Moreover, IsoKs have been detected in brain and nervous tissue as a consequence of oxidative damage. The cells of these tissues meet two conditions for preferential IsoK-PE adduct formation: they are rich in AA acyl chains, which are a source for isoketal formation, and in PE [97,99,119]. This makes an investigation of the mechanisms by which PE adducts influence the function of membrane proteins very important.

## References

[1] Skulachev V.P. (2012). Mitochondria-Targeted Antioxidants as Promising Drugs for Treatment of Age-Related Brain Diseases. J. Alzheimers Dis..

[2] Wong H.S., Dighe P.A., Mezera V., Monternier P.A., Brand M.D. (2017). Production of Superoxide and Hydrogen Peroxide from Specific Mitochondrial Sites under Different Bioenergetic Conditions. J. Biol. Chem..

[3] Brand M.D. (2016). Mitochondrial Generation of Superoxide and Hydrogen Peroxide as the Source of Mitochondrial Redox Signaling. Free Radic. Biol. Med..

[4] Murphy M. (2016). Mitochondrial Superoxide Production in Health and Disease. Biochim. Biophys. Acta.

[5] Vinogradov A.D., Grivennikova V.G. (2016). Oxidation of Nadh And Ros Production By Respiratory Complex, I. Biochim. Biophys. Acta.

[6] Naudi A., Jove M., Ayala V., Cabre R., Portero-Otin M., Pamplona R. (2013). Non-Enzymatic Modification of Aminophospholipids by Carbonyl-Amine Reactions. Int. J. Mol. Sci..

[7] Esterbauer H., Schaur R.J., Zollner H. (1991). Chemistry and Biochemistry Of 4-Hydroxynonenal, Malonaldehyde and Related Aldehydes. Free Radic. Biol. Med..

[8] Roede J.R., Jones D.P. (2010). Reactive Species and Mitochondrial Dysfunction: Mechanistic Significance of 4-Hydroxynonenal. Environ Mol Mutagen.

[9] Zarkovic N. (2003). 4-Hydroxynonenal as a Bioactive Marker of Pathophysiological Processes. Mol. Asp. Med..

[10] Kasai H., Maekawa M., Kawai K., Hachisuka K., Takahashi Y., Nakamura H., Sawa R., Matsui S., Matsuda T. (2005). 4-Oxo-2-Hexenal, A Mutagen Formed By Omega-3 Fat Peroxidation, Causes Dna Adduct Formation In Mouse Organs. Ind Health.

[11] Lin D., Lee H.G., Liu Q., Perry G., Smith M.A., Sayre L.M. (2005). 4-Oxo-2-Nonenal Is Both More Neurotoxic And More Protein Reactive Than 4-Hydroxy-2-Nonenal. Chem. Res. Toxicol..

[12] Valko M., Leibfritz D., Moncol J., Cronin M.T., Mazur M., Telser J. (2007). Free Radicals And Antioxidants In Normal Physiological Functions And Human Disease. Int. J. Biochem. Cell Biol..

[13] Benedetti A., Comporti M., Fulceri R., Esterbauer H. (1984). Cytotoxic Aldehydes Originating from the Peroxidation OF Liver Microsomal Lipids. Identification Of 4,5-Dihydroxydecenal. Biochim. Biophys. Acta.

[14] Uchida K. (2003). 4-Hydroxy-2-Nonenal: A Product and Mediator of Oxidative Stress. Prog. Lipid Res..

[15] Koster J.F., Slee R.G., Montfoort A., Lang J., Esterbauer H. (1986). Comparison of The Inactivation of Microsomal Glucose-6-Phosphatase By In Situ Lipid Peroxidation-Derived 4-Hydroxynonenal And Exogenous 4-Hydroxynonenal. Free Radic. Res. Commun..

[16] Jovanovic O., Pashkovskaya A.A., Annibal A., Vazdar M., Burchardt N., Sansone A., Gille L., Fedorova M., Ferreri C., Pohl E.E. (2015). The Molecular Mechanism behind Reactive Aldehyde Action on Transmembrane Translocations of Proton and Potassium Ions. Free Radic. Biol. Med..

[17] Droge W. (2002). Free Radicals in the Physiological Control of Cell Function. Physiol. Rev..

[18] Hannesschlaeger C., Pohl P. (2018). Membrane Permeabilities of Ascorbic Acid and Ascorbate. Biomolecules.

[19] Lushchak V.I. (2014). Free Radicals, Reactive Oxygen Species, Oxidative Stress and Its Classification. Chem. Biol. Interact..

[20] Tikhonova I.M., Andreyev A.Y., Kaulen A.D., Komrakov A.Y., Skulachev V.P. (1994). Ion Permeability Induced In Artificial Membranes By The Atp/Adp Antiporter. FEBS Lett..

[21] Bertholet A.M., Chouchani E.T., Kazak L., Angelin A., Fedorenko A., Long J.Z., Vidoni S., Garrity R., Cho J., Terada N. (2019). H(+) Transport Is An Integral Function of The Mitochondrial Adp/Atp Carrier. Nature.

[22] Wieckowski M.R., Wojtczak L. (1997). Involvement of the Dicarboxylate Carrier in the Protonophoric Action of Long-Chain Fatty Acids In Mitochondria. Biochem. Biophys. Res. Commun..

[23] Samartsev V.N., Marchik E.I., Shamagulova L.V. (2011). Free Fatty Acids As Inducers And Regulators Of Uncoupling Of Oxidative Phosphorylation In Liver Mitochondria With Participation Of Adp/Atp- And Aspartate/Glutamate-Antiporter. Biochemistry.

[24] Shabalina I.G., Jacobsson A., Cannon B., Nedergaard J. (2004). Native Ucp1 Displays Simple Competitive Kinetics between the Regulators Purine Nucleotides and Fatty Acids. J. Biol. Chem..

[25] Urbankova E., Voltchenko A., Pohl P., Jezek P., Pohl E.E. (2003). Transport Kinetics Of Uncoupling Proteins. Analysis of Ucp1 Reconstituted In Planar Lipid Bilayers. J. Biol. Chem..

[26] Macher G., Koehler M., Rupprecht A., Kreiter J., Hinterdorfer P., Pohl E.E. (2018). Inhibition Of Mitochondrial Ucp1 And Ucp3 By Purine Nucleotides And Phosphate. Biochim. Biophys. Acta Biomembr..

[27] Jezek P., Orosz D.E., Garlid K.D. (1990). Reconstitution of the Uncoupling Protein of Brown Adipose Tissue Mitochondria. Demonstration of Gdp-Sensitive Halide Anion Uniport. J. Biol. Chem..

[28] Bouillaud F., Ricquier D., Gulik-Krzywicki T., Gary-Bobo C.M. (1983). The Possible Proton Translocating Activity of the Mitochondrial Uncoupling Protein of Brown Adipose Tissue. Reconstitution Studies in Liposomes. FEBS Lett..

[29] Bienengraeber M., Echtay K.S., Klingenberg M. (1998). H^+^ Transport by Uncoupling Protein (Ucp-1) Is Dependent On A Histidine Pair, Absent In Ucp-2 and Ucp-3. Biochemistry.

[30] Cannon B., Shabalina I.G., Kramarova T.V., Petrovic N., Nedergaard J. (2006). Uncoupling Proteins: A Role In Protection Against Reactive Oxygen Species-Or Not?. Biochim. Biophys. Acta.

[31] Pohl E.E., Rupprecht A., Macher G., Hilse K.E. (2019). Important Trends In Ucp3 Investigation. Front. Physiol..

[32] Vozza A., Parisi G., De Leonardis F., Lasorsa F.M., Castegna A., Amorese D., Marmo R., Calcagnile V.M., Palmieri L., Ricquier D. (2014). Ucp2 Transports C4 Metabolites Out Of Mitochondria, Regulating Glucose And Glutamine Oxidation. Proc. Natl. Acad. Sci. USA.

[33] Rupprecht A., Moldzio R., Modl B., Pohl E.E. (2019). Glutamine Regulates Mitochondrial Uncoupling Protein 2 To Promote Glutaminolysis In Neuroblastoma Cells. Biochim. Biophys. Acta Bioenerg..

[34] Hilse K.E., Rupprecht A., Egerbacher M., Bardakji S., Zimmermann L., Wulczyn A., Pohl E.E. (2018). The Expression Of Uncoupling Protein 3 Coincides With The Fatty Acid Oxidation Type Of Metabolism In Adult Murine Heart. Front. Physiol..

[35] Beck V., Jaburek M., Demina T., Rupprecht A., Porter R.K., Jezek P., Pohl E.E. (2007). Polyunsaturated Fatty Acids Activate Human Uncoupling Proteins 1 And 2 In Planar Lipid Bilayers. FASEB J..

[36] Zackova M., Jezek P. (2002). Reconstitution of Novel Mitochondrial Uncoupling Proteins Ucp2 and Ucp3. Biosci. Rep..

[37] Zackova M., Skobisova E., Urbankova E., Jezek P. (2003). Activating Omega-6 Polyunsaturated Fatty Acids And Inhibitory Purine Nucleotides Are High Affinity Ligands For Novel Mitochondrial Uncoupling Proteins Ucp2 And Ucp3. J. Biol. Chem..

[38] Rupprecht A., Sittner D., Smorodchenko A., Hilse K.E., Goyn J., Moldzio R., Seiler A.E., Brauer A.U., Pohl E.E. (2014). Uncoupling Protein 2 And 4 Expression Pattern During Stem Cell Differentiation Provides New Insight Into Their Putative Function. PLoS ONE.

[39] Hilse K.E., Kalinovich A.V., Rupprecht A., Smorodchenko A., Zeitz U., Staniek K., Erben R.G., Pohl E.E. (2016). The Expression Of Ucp3 Directly Correlates To Ucp1 Abundance In Brown Adipose Tissue. Biochim. Biophys. Acta.

[40] Negre-Salvayre A., Hirtz C., Carrera G., Cazenave R., Troly M., Salvayre R., Penicaud L., Casteilla L. (1997). A Role for Uncoupling Protein-2 as a Regulator of Mitochondrial Hydrogen Peroxide Generation. Faseb, J..

[41] Echtay K.S., Roussel D., St-Pierre J., Jekabsons M.B., Cadenas S., Stuart J.A., Harper J.A., Roebuck S.J., Morrison A., Pickering S. (2002). Superoxide Activates Mitochondrial Uncoupling Proteins. Nature.

[42] Echtay K.S., Esteves T.C., Pakay J.L., Jekabsons M.B., Lambert A.J., Portero-Otin M., Pamplona R., Vidal-Puig A.J., Wang S., Roebuck S.J. (2003). A Signalling Role for 4-Hydroxy-2-Nonenal in Regulation of Mitochondrial Uncoupling. EMBO J..

[43] Krauss S., Zhang C.Y., Lowell B.B. (2005). The Mitochondrial Uncoupling-Protein Homologues. Nat. Rev. Mol. Cell Biol..

[44] Shabalina I.G., Petrovic N., Kramarova T.V., Hoeks J., Cannon B., Nedergaard J. (2006). Ucp1 And Defense Against Oxidative Stress. 4-Hydroxy-2-Nonenal Effects On Brown Fat Mitochondria Are Uncoupling Protein 1-Independent. J. Biol. Chem..

[45] Couplan E., Mar Gonzalez-Barroso M., Alves-Guerra M.C., Ricquier D., Goubern M., Bouillaud F. (2002). No Evidence for A Basal, Retinoic, or Superoxide-Induced Uncoupling Activity of the Uncoupling Protein 2 Present In Spleen or Lung Mitochondria. J. Biol.Chem..

[46] Lombardi A., Grasso P., Moreno M., De Lange P., Silvestri E., Lanni A., Goglia F. (2008). Interrelated Influence Of Superoxides And Free Fatty Acids Over Mitochondrial Uncoupling In Skeletal Muscle. Biochim. Biophys. Acta.

[47] Parker N., Vidal-Puig A., Brand M.D. (2008). Stimulation of Mitochondrial Proton Conductance by Hydroxynonenal Requires A High Membrane Potential. Biosci. Rep..

[48] Malingriaux E.A., Rupprecht A., Gille L., Jovanovic O., Jezek P., Jaburek M., Pohl E.E. (2013). Fatty Acids Are Key In 4-Hydroxy-2-Nonenal-Mediated Activation Of Uncoupling Proteins 1 And 2. PLoS ONE.

[49] Zarkovic N., Cipak A., Jaganjac M., Borovic S., Zarkovic K. (2013). Pathophysiological Relevance of Aldehydic Protein Modifications. J. Proteomics..

[50] Fritz K.S., Petersen D.R. (2013). An Overview of the Chemistry and Biology Of Reactive Aldehydes. Free Radic. Biol. Med..

[51] Castro J.P., Jung T., Grune T., Siems W. (2017). 4-Hydroxynonenal (Hne) Modified Proteins in Metabolic Diseases. Free Radic. Biol. Med..

[52] Voulgaridou G.P., Anestopoulos I., Franco R., Panayiotidis M.I., Pappa A. (2011). Dna Damage Induced By Endogenous Aldehydes: Current State Of Knowledge. Mutat. Res..

[53] Gentile F., Arcaro A., Pizzimenti S., Daga M., Cetrangolo G.P., Dianzani C., Lepore A., Graf M., Ames P.R.J., Barrera G. (2017). Dna Damage By Lipid Peroxidation Products: Implications In Cancer, Inflammation And Autoimmunity. AIMS Genet..

[54] Guo L., Davies S.S. (2013). Bioactive Aldehyde-Modified Phosphatidylethanolamines. Biochimie.

[55] Sousa B.C., Pitt A.R., Spickett C.M. (2017). Chemistry and Analysis of Hne and Other Prominent Carbonyl-Containing Lipid Oxidation Compounds. Free Radic. Biol. Med..

[56] Sokolov V.S., Batishchev O.V., Akimov S.A., Galimzyanov T.R., Konstantinova A.N., Malingriaux E., Gorbunova Y.G., Knyazev D.G., Pohl P. (2018). Residence Time Of Singlet Oxygen In Membranes. Sci. Rep..

[57] Gueraud F., Atalay M., Bresgen N., Cipak A., Eckl P.M., Huc L., Jouanin I., Siems W., Uchida K. (2010). Chemistry And Biochemistry Of Lipid Peroxidation Products. Free Radic. Res..

[58] Doorn J.A., Petersen D.R. (2002). Covalent Modification of Amino Acid Nucleophiles by the Lipid Peroxidation Products 4-Hydroxy-2-Nonenal and 4-Oxo-2-Nonenal. Chem. Res. Toxicol..

[59] Carbone D.L., Doorn J.A., Kiebler Z., Petersen D.R. (2005). Cysteine Modification by Lipid Peroxidation Products Inhibits Protein Disulfide Isomerase. Chem. Res. Toxicol..

[60] Brame C.J., Salomon R.G., Morrow J.D., Roberts L.J. (1999). Identification Of Extremely Reactive Gamma-Ketoaldehydes (Isolevuglandins) As Products Of The Isoprostane Pathway And Characterization of Their Lysyl Protein Adducts. J. Biol. Chem..

[61] Bleier L., Wittig I., Heide H., Steger M., Brandt U., Drose S. (2015). Generator-Specific Targets Of Mitochondrial Reactive Oxygen Species. Free Radic. Biol. Med..

[62] Bogeski I., Kappl R., Kummerow C., Gulaboski R., Hoth M., Niemeyer B.A. (2011). Redox Regulation of Calcium Ion Channels: Chemical And Physiological Aspects. Cell Calcium.

[63] Van Der Veen J.N., Kennelly J.P., Wan S., Vance J.E., Vance D.E., Jacobs R.L. (2017). The Critical Role of Phosphatidylcholine and Phosphatidylethanolamine Metabolism in Health and Disease. Biochim. Biophys. Acta Biomembr..

[64] Vance J.E., Tasseva G. (2013). Formation and Function of Phosphatidylserine and Phosphatidylethanolamine in Mammalian Cells. Biochim. Biophys. Acta.

[65] Devaux P.F., Morris R. (2004). Transmembrane Asymmetry and Lateral Domains in Biological Membranes. Traffic.

[66] Epand R.M., Fuller N., Rand R.P. (1996). Role of the Position of Unsaturation on The Phase Behavior And Intrinsic Curvature Of Phosphatidylethanolamines. Biophys. J..

[67] Van Den Brink-Van Der Laan E., Killian J.A., De Kruijff B. (2004). Nonbilayer Lipids Affect Peripheral and Integral Membrane Proteins via Changes in the Lateral Pressure Profile. Biochim. Biophys. Acta.

[68] Cullis P.R., De Kruijff B. (1979). Lipid Polymorphism and the Functional Roles of Lipids in Biological Membranes. Biochim. Biophys. Acta.

[69] Verkleij A.J., Leunissen-Bijvelt J., De Kruijff B., Hope M., Cullis P.R. (1984). Non-Bilayer Structures in Membrane Fusion. Ciba Found. Symp..

[70] Siegel D.P., Epand R.M. (1997). The Mechanism of Lamellar-To-Inverted Hexagonal Phase Transitions in Phosphatidylethanolamine: Implications for Membrane Fusion Mechanisms. Biophys. J..

[71] Martens C., Shekhar M., Borysik A.J., Lau A.M., Reading E., Tajkhorshid E., Booth P.J., Politis A. (2018). Direct Protein-Lipid Interactions Shape The Conformational Landscape Of Secondary Transporters. Nat. Commun..

[72] Van Den Brink-Van Der Laan E., Chupin V., Killian J.A., De Kruijff B. (2004). Stability of Kcsa Tetramer Depends On Membrane Lateral Pressure. Biochemistry.

[73] Vance J.E. (2015). Phospholipid Synthesis and Transport in Mammalian Cells. Traffic.

[74] Bogdanov M., Dowhan W. (1999). Lipid-Assisted Protein Folding. J. Biol. Chem..

[75] Shinzawa-Itoh K., Aoyama H., Muramoto K., Terada H., Kurauchi T., Tadehara Y., Yamasaki A., Sugimura T., Kurono S., Tsujimoto K. (2007). Structures And Physiological Roles Of 13 Integral Lipids Of Bovine Heart Cytochrome C Oxidase. EMBO J..

[76] Calzada E., Avery E., Sam P.N., Modak A., Wang C., Mccaffery J.M., Han X., Alder N.N., Claypool S.M. (2019). Phosphatidylethanolamine Made In The Inner Mitochondrial Membrane Is Essential For Yeast Cytochrome Bc1 Complex Function. Nat. Commun..

[77] Ichimura Y., Kirisako T., Takao T., Satomi Y., Shimonishi Y., Ishihara N., Mizushima N., Tanida I., Kominami E., Ohsumi M. (2000). A Ubiquitin-Like System Mediates Protein Lipidation. Nature.

[78] Kagan V.E., Mao G., Qu F., Angeli J.P., Doll S., Croix C.S., Dar H.H., Liu B., Tyurin V.A., Ritov V.B. (2017). Oxidized Arachidonic And Adrenic Pes Navigate Cells To Ferroptosis. Nat. Chem. Biol..

[79] Deleault N.R., Piro J.R., Walsh D.J., Wang F., Ma J., Geoghegan J.C., Supattapone S. (2012). Isolation Of Phosphatidylethanolamine As A Solitary Cofactor For Prion Formation In The Absence Of Nucleic Acids. Proc. Natl. Acad. Sci. USA.

[80] Menon A.K., Eppinger M., Mayor S., Schwarz R.T. (1993). Phosphatidylethanolamine Is the Donor of the Terminal Phosphoethanolamine Group in Trypanosome Glycosylphosphatidylinositols. EMBO J..

[81] Vance J.E. (2018). Historical Perspective: Phosphatidylserine and Phosphatidylethanolamine from the 1800s to the Present. J. Lipid Res..

[82] Calzada E., Onguka O., Claypool S.M. (2016). Phosphatidylethanolamine Metabolism in Health And Disease. Int. Rev. Cell Mol. Biol..

[83] Chidley C., Trauger S.A., Birsoy K., O’shea E.K. (2016). The Anticancer Natural Product Ophiobolin A Induces Cytotoxicity By Covalent Modification of Phosphatidylethanolamine. Elife.

[84] Guichardant M., Taibi-Tronche P., Fay L.B., Lagarde M. (1998). Covalent Modifications of Aminophospholipids by 4-Hydroxynonenal. Free Radic. Biol. Med..

[85] Bacot S., Bernoud-Hubac N., Baddas N., Chantegrel B., Deshayes C., Doutheau A., Lagarde M., Guichardant M. (2003). Covalent Binding Of Hydroxy-Alkenals 4-Hdde, 4-Hhe, And 4-Hne To Ethanolamine Phospholipid Subclasses. J. Lipid Res..

[86] Guo L., Amarnath V., Davies S.S. (2010). A Liquid Chromatography-Tandem Mass Spectrometry Method for Measurement of *N*-Modified Phosphatidylethanolamines. Anal. Biochem..

[87] Annibal A., Schubert K., Wagner U., Hoffmann R., Schiller J., Fedorova M. (2014). New Covalent Modifications Of Phosphatidylethanolamine By Alkanals: Mass Spectrometry Based Structural Characterization And Biological Effects. J. Mass Spectrom..

[88] Vazdar K., Vojta D., Margetic D., Vazdar M. (2017). Reaction Mechanism of Covalent Modification of Phosphatidylethanolamine Lipids by Reactive Aldehydes 4-Hydroxy-2-Nonenal And 4-Oxo-2-Nonenal. Chem. Res. Toxicol..

[89] Jovanovic O., Skulj S., Pohl E.E., Vazdar M. (2019). Covalent Modification of Phosphatidylethanolamine by 4-Hydroxy-2-Nonenal Increases Sodium Permeability across Phospholipid Bilayer Membranes. Free Radic. Biol. Med..

[90] Annibal A., Riemer T., Jovanovic O., Westphal D., Griesser E., Pohl E.E., Schiller J., Hoffmann R., Fedorova M. (2016). Structural, Biological And Biophysical Properties of Glycated and Glycoxidized Phosphatidylethanolamines. Free Radic. Biol. Med..

[91] Guo L., Chen Z., Amarnath V., Davies S.S. (2012). Identification Of Novel Bioactive Aldehyde-Modified Phosphatidylethanolamines Formed By Lipid Peroxidation. Free Radic. Biol.Med..

[92] Stadelmann-Ingrand S., Favreliere S., Fauconneau B., Mauco G., Tallineau C. (2001). Plasmalogen Degradation by Oxidative Stress: Production and Disappearance of Specific Fatty Aldehydes and Fatty Alpha-Hydroxyaldehydes. Free Radic. Biol. Med..

[93] Stadelmann-Ingrand S., Pontcharraud R., Fauconneau B. (2004). Evidence For The Reactivity Of Fatty Aldehydes Released From Oxidized Plasmalogens With Phosphatidylethanolamine To Form Schiff Base Adducts In Rat Brain Homogenates. Chem. Phys. Lipids.

[94] Guo L., Chen Z., Cox B.E., Amarnath V., Epand R.F., Epand R.M., Davies S.S. (2011). Phosphatidylethanolamines Modified By Gamma-Ketoaldehyde (Gammaka) Induce Endoplasmic Reticulum Stress And Endothelial Activation. J. Biol. Chem..

[95] Bernoud-Hubac N., Fay L.B., Armarnath V., Guichardant M., Bacot S., Davies S.S., Roberts II L.J., Lagarde M. (2004). Covalent Binding Of Isoketals to Ethanolamine Phospholipids. Free Radic. Biol. Med..

[96] Sullivan C.B., Matafonova E., Roberts II L.J., Amarnath V., Davies S.S. (2010). Isoketals Form Cytotoxic Phosphatidylethanolamine Adducts In Cells. J. Lipid Res..

[97] Guo L., Chen Z., Amarnath V., Yancey P.G., Van Lenten B.J., Savage J.R., Fazio S., Linton M.F., Davies S.S. (2015). Isolevuglandin-Type Lipid Aldehydes Induce The Inflammatory Response Of Macrophages By Modifying Phosphatidylethanolamines And Activating The Receptor For Advanced Glycation Endproducts. Antioxid. Redox Signal..

[98] Davies S.D., May-Zhang L.S., Boutaud O., Amarnath V., Kirabo A., Harrison D.G. (2019). Isolevuglandins as Mediators of Disease and the Development of Dicarbonyl Scavengers as Pharmaceutical Interventions. Pharmacol. Ther.

[99] Salomon R.G., Bi W. (2015). Isolevuglandin Adducts In Disease. Antioxid. Redox Signal..

[100] May-Zhang L.S., Yermalitsky V., Huang J., Pleasent T., Borja M.S., Oda M.N., Jerome W.G., Yancey P.G., Linton M.F., Davies S.S. (2018). Modification By Isolevuglandins, Highly Reactive Gamma-Ketoaldehydes, Deleteriously Alters High-Density Lipoprotein Structure And Function. J. Biol. Chem..

[101] Zemski Berry K.A., Murphy R.C. (2007). Characterization of Acrolein-Glycerophosphoethanolamine Lipid Adducts Using Electrospray Mass Spectrometry. Chem. Res. Toxicol..

[102] Ravandi A., Kuksis A., Marai L., Myher J.J. (1995). Preparation and Characterization of Glucosylated Aminoglycerophospholipids. Lipids.

[103] Fountain W.C., Requena J.R., Jenkins A.J., Lyons T.J., Smyth B., Baynes J.W., Thorpe S.R. (1999). Quantification of N-(Glucitol)Ethanolamine and N-(Carboxymethyl)Serine: Two Products of Nonenzymatic Modification of Aminophospholipids Formed in Vivo. Anal. Biochem..

[104] Requena J.R., Ahmed M.U., Fountain C.W., Degenhardt T.P., Reddy S., Perez C., Lyons T.J., Jenkins A.J., Baynes J.W., Thorpe S.R. (1997). Carboxymethylethanolamine, A Biomarker Of Phospholipid Modification During The Maillard Reaction In Vivo. J. Biol. Chem..

[105] Lapolla A., Fedele D., Traldi P. (2005). Glyco-Oxidation in Diabetes and Related Diseases. Clin. Chim. Acta.

[106] Simoes C., Silva A.C., Domingues P., Laranjeira P., Paiva A., Domingues M.R. (2013). Modified Phosphatidylethanolamines Induce Different Levels Of Cytokine Expression In Monocytes And Dendritic Cells. Chem. Phys. Lipids.

[107] Van Meer G., Voelker D.R., Feigenson G.W. (2008). Membrane Lipids: Where they are and How They Behave. Nat. Rev. Mol. Cell Biol..

[108] Patel D., Witt S.N. (2017). Ethanolamine and Phosphatidylethanolamine: Partners in Health and Disease. Oxid. Med. Cell Longev..

[109] Peterson U., Mannock D.A., Lewis R.N., Pohl P., Mcelhaney R.N., Pohl E.E. (2002). Origin of Membrane Dipole Potential: Contribution of The Phospholipid Fatty Acid Chains. Chem. Phys. Lipids.

[110] Hannesschlaeger C., Horner A., Pohl P. (2019). Intrinsic Membrane Permeability to Small Molecules. Chem. Rev..

[111] Chekashkina K., Jovanovic O., Kuzmin P., Pohl E., Pavel B. (2017). The Changes of Physical Parameters of Lipid Membrane Caused By Lipid Peroxidation-Derived Aldehydes. Biophys. J..

[112] Galimzyanov T.R., Kuzmin P.I., Pohl P., Akimov S.A. (2017). Undulations Drive Domain Registration From The Two Membrane Leaflets. Biophys. J..

[113] Jovanovic O., Chekashkina K., Bashkirov P., Vazdar M., Pohl E.E. (2017). Uncoupling Proteins Are Highly Sensitive to the Membrane Lipid Composition. Eur. Biophys. J. Biophys. Lett..

[114] Pohl P., Rokitskaya T.I., Pohl E.E., Saparov S.M. (1997). Permeation Of Phloretin Across Bilayer Lipid Membranes Monitored By Dipole Potential And Microelectrode Measurements. Biochim. Biophys. Acta.

[115] Pohl E.E., Krylov A.V., Block M., Pohl P. (1998). Changes of the Membrane Potential Profile Induced By Verapamil and Propranolol. Biochim. Biophys. Acta.

[116] Jovanovic O., Chekashkina K., Bashkirov P., Skuljc S., Vazdar M., Pohl E.E. (2019). Lipid Curvature Modulates Function of Mitochondrial Membrane Proteins. Eur. Biophys. J. Biophys. Lett..

[117] Aimon S., Callan-Jones A., Berthaud A., Pinot M., Toombes G.E., Bassereau P. (2014). Membrane Shape Modulates Transmembrane Protein Distribution. Dev. Cell.

[118] Phillips R., Ursell T., Wiggins P., Sens P. (2009). Emerging Roles for Lipids in Shaping Membrane-Protein Function. Nature.

[119] Farooqui A.A., Horrocks L.A., Farooqui T. (2007). Modulation of Inflammation in Brain: A Matter of Fat. J. Neurochem..

